# Simvastatin Posttreatment Controls Inflammation and Improves Bacterial Clearance in Experimental Sepsis

**DOI:** 10.1155/2020/1839762

**Published:** 2020-10-14

**Authors:** Flora Magno de Jesus Oliveira, Cassiano Felippe Gonçalves-de-Albuquerque, Isabel Matos Medeiros de Moraes, Patrícia Alves Reis, Vinicius Novaes Rocha, Patrícia Torres Bozza, Adriana Ribeiro Silva, Hugo Caire de Castro Faria Neto

**Affiliations:** ^1^Laboratório de Imunofarmacologia, Instituto Oswaldo Cruz, Fiocruz, Rio de Janeiro, RJ, Brazil; ^2^Laboratório de Imunofarmacologia, Instituto Biomédico, Universidade Federal do Estado do Rio de Janeiro, Brazil; ^3^Laboratório de Patologia e Histologia Veterinária, Departamento de Medicina Veterinária, Universidade Federal de Juiz de Fora, Brazil

## Abstract

Sepsis is characterized by a life-threatening organ dysfunction caused by an unbalanced host response to microbe infection that can lead to death. Besides being currently the leading cause of death in intensive care units worldwide, sepsis can also induce long-term consequences among survivors, such as cognitive impairment. Statins (lipid-lowering drugs widely used to treat dyslipidemia) have been shown to possess pleiotropic anti-inflammatory and antimicrobial effects. These drugs act inhibiting 3-hydroxy-3-methylglutaryl-coenzyme A (HMG-CoA) reductase, an enzyme that catalyzes the conversion of HMG-CoA to mevalonate, the limiting step in cholesterol biosynthesis. In this work, we evaluated the therapeutic effects of simvastatin in an animal model of sepsis. In previous study from our group, statin pretreatment avoided cognitive damage and neuroinflammation in sepsis survivors. Herein, we focused on acute inflammation where sepsis was induced by cecal ligation and puncture (CLP), and the animals were treated with simvastatin (2 mg/kg) 6 h after surgery. We measured plasma biochemical markers of organ dysfunction, cell migration, cell activation, bacterial elimination, production of nitric oxide 24 h after CLP, survival rate for 7 days, and cognitive impairment 15 days after CLP. One single administration of simvastatin 6 h after CLP was able to prevent both liver and kidney dysfunction. In addition, this drug decreased cell accumulation in the peritoneum as well as the levels of TNF-*α*, MIF, IL-6, and IL-1*β*. Simvastatin diminished the number of bacterial colony forming units (CFU) and increased the production of nitric oxide production in the peritoneum. Simvastatin treatment increased survival for the first 24 h, but it did not alter survival rate at the end of 7 days. Our results showed that posttreatment with simvastatin hampered organ dysfunction, increased local production of nitric oxide, improved bacterial clearance, and modulated inflammation in a relevant model of sepsis.

## 1. Introduction

According to the Third International Consensus, sepsis is defined as life-threatening organ dysfunction caused by a deregulated host response to infection [1]. Sepsis is one of the most common causes of death and critical illness in the world and is increasingly prevalent in the developed world with high financial cost. Furthermore, systemic infection is often revealed by or associated with brain dysfunction, which is characterized by alteration of consciousness, ranging from delirium to coma, seizure or focal neurological signs, and long-term cognitive disability [2]. Sepsis evolves when the host cannot limit primary infection, leading to a severe inflammatory response syndrome (SIRS) [3] that can be followed by immunosuppression [4].

Statins are lipid lowering drugs, indicated for the prevention of cardiovascular diseases [5]. Statins compete with and inhibit the enzyme HMG-CoA reductase, hindering cholesterol and isoprenoids synthesis which ends up affecting protein-prenylation that impacts, for instance, on Rho and Rac kinase pathways [6, 7]. For a long time, the effects of statins on cholesterol homeostasis have been attributed to the inhibition of isoprenylations and farnesilations of intracellular kinases. In past few years, the growing interest in statin use as potential inhibitors of inflammation [8] widened the clinical potential of these drugs. Statins alter the availability of cholesterol, the vascular inflammatory response, chemotaxis [9], decrease oxidative stress and production of superoxide anions in blood from septic patients [10], and present antimicrobial effects [11, 12]. All effects may be due, at least in part, to the alteration of protein-prenylation.

During sepsis, statins affect the production of IL-6, IL-8, TNF, MCP-1, and C-reactive protein [13]. Furthermore, simvastatin [14] and cerivastatin improved survival rate and reduced serum TNF-*α* and IL-1*β* in a murine model of sepsis [15]. Our group has demonstrated important pleiotropic effects of pretreatment with statins including inhibition of neuroinflammation and cerebral microcirculatory dysfunction in models of sepsis, malaria and hypertension [6, 16, 17]. These studies provided significant evidence supporting the immunomodulatory effects of statins in inflammatory conditions. In the present work, we investigated the effects of simvastatin given 6 h after the induction of sepsis by CLP. We analyzed markers of organ dysfunction, inflammatory parameters, and bacterial clearance focusing on the acute phase of inflammatory response after sepsis.

## 2. Materials and Methods

### 2.1. Animals

Male Swiss Webster (SW) mice, weighing 20 to 30 g, were obtained from Oswaldo Cruz Foundation breeding unit. All the animals were maintained at constant temperature of 22°C, with 12 hours light/dark cycle, and had ad libitum access to standard chow and water. The experimental procedures described in this work were approved by the Institutional Animal Welfare Committee (CEUA-Fiocruz # 0260-05). Our Institution follow the ARRIVE guidelines (Animal Research: Reporting of In Vivo Experiments) originally published in 2010 [18].

### 2.2. Sepsis Induction and Treatment

Polymicrobial sepsis was induced by CLP performed as previously described [19]. Briefly, mice were anesthetized with a mixture of ketamine (100 mg/kg) and xylazine (10 mg/kg) diluted in sterile saline and administered intraperitoneally (0.2 ml). After aseptic procedures with 70% ethanol, an incision was made through the *linea alba*. The cecum was exposed, ligated with sterile 3-0 silk immediately after the ileum-cecal valve in a way to avoid obstruction of intestinal transit, and subjected to two through-and-through perforations with 18-gauge needle. A small amount of fecal material was expelled into peritoneal cavity, and the cecum was gently relocated. The area was sutured with nylon 3-0 (Shalon) in two layers. Sham-operated mice were submitted to the same procedure except for the ligation and perforation of the cecum. All mice received a volume support of 1 ml prewarmed sterile saline by subcutaneously route immediately after surgery.

A single dose of simvastatin (Sigma-Aldrich, 2 mg/kg) or vehicle (0.2% DMSO in saline) was administered intravenously, through the orbital plexus, six hours after sepsis induction. All animals were treated with antibiotic (imipenem/cilastatin, 10 mg/kg body weight) six hours after the surgery.

Twenty-four hours after sepsis induction, the animals had their peritoneal cavity opened and washed with 3 ml of sterile saline. The peritoneal lavage was collected for total and differential cell count, colony forming unit evaluation, cytokine analysis, lipid body quantification, and nitric oxide (NO) dosage. Survival rate was observed daily during 7 days.

### 2.3. Leukocyte Count

Peritoneal lavage samples were diluted in Turk (2% acetic acid), and the total cell counts were performed with optical microscopy in Neubauer chamber. For differential cell count, the samples were cytocentrifuged in a microscope slide and stained with Panoptic fast Kit.

### 2.4. Lipid Body Staining and Counting

Peritoneal lavage samples were cytocentrifuged and fixed in 3.7% formaldehyde at room temperature. The cells were stained by osmium tetroxide, as previously described [20]. Lipid bodies were enumerated by optical microscopy in 50 consecutive leukocytes.

### 2.5. Cytokine Measurement

IL-6, TNF-*α*, IL-1*β*, and MIF were measured in cell-free peritoneal fluid supernatants using ELISA kits following the manufacturer's instructions (Duo Set, R&D Systems, Minneapolis, USA).

### 2.6. Quantification of Colony Forming Units

The peritoneal lavage from each animal was diluted and plated on tryptic soy agar plates. After 24 hours of incubation at 37°C, the number of bacterial colonies was determined manually.

For *in vitro* experiments, naïve SW mice were treated by intraperitoneal route with 3 ml of thioglycolate. After 3 days, they were euthanized, and macrophages were collected by peritoneal lavage. Peritoneal macrophages were then cultured and treated with simvastatin at three different concentrations (5 *μ*M, 10 *μ*M, 20 *μ*M). After 15 minutes, the cells were incubated with *Escherichia coli* (*E.coli)* (10^5^ bacteria/ml) for 30 minutes. The supernatant was collected and plated for CFU quantification.

### 2.7. Nitric Oxide Quantification

NO was indirectly determined using the Griess method [21]. In brief, plasma samples of each animal obtained by cardiac puncture and peritoneal fluid supernatants were added to Griess reagent. The absorbance at 550 nm was measured, and the nitrite concentration was determined compared to a nitrite standard curve.

### 2.8. Biochemistry Analysis

Twenty-four hours after sepsis induction, a group of animals was euthanized, and blood samples were collected by cardiac puncture. The biochemistry analyses were done at FIOCRUZ core facility service through a dry chemistry method.

### 2.9. Histological Analysis

Liver and kidney tissues were isolated from mice and immediately fixed in 10% phosphate-buffered formalin. Tissues were processed and embedded in paraffin, and sections of 5 *μ*M were routinely stained with hematoxylin & eosin.

### 2.10. Step-Down Inhibitory Avoidance Test

The step-down inhibitory avoidance test was performed as we previously described [22]. In the training trial, animals were placed on a platform, and their latency to step down on the grid with all four paws was measured with an automatic device. Immediately upon stepping down on the grid, the animals received a 0.6 mA, 3.0-second foot shock. A retention test trial was performed 1.5 and 24 hours after training, and latency to step on the grid was recorded.

### 2.11. Statistical Analysis

Results were analyzed by ANOVA, followed by the Newman-Keuls test. All data are expressed as mean ± SEM, and a significance value was established as *p* < 0.05. The survival rate was analyzed by the Long-rank test.

## 3. Results

### 3.1. Simvastatin Improved Renal and Hepatic Function in Septic Animals and Decreased Cell Accumulation and Cytokine Levels in the Peritoneal Lavage of Septic Animals

**Figure 1:**
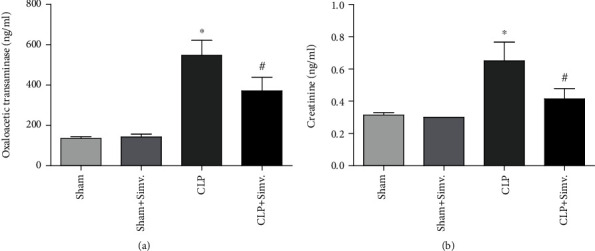
Simvastatin effect on hepatic (a) and renal (b) function in the animals submitted to CLP. SW mice submitted to CLP, treated or untreated with simvastatin, had the blood collected through cardiac puncture 24 hours after surgery for biochemistry analysis. Data represented as mean ± SEM of at least 6 animals. ^∗^*p* < 0.05, control vs. CLP + vehicle; ^#^*p* < 0.05, CLP + vehicle vs. CLP + simvastatin.

**Figure 2:**
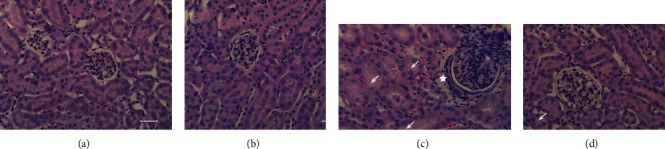
Representative histology photomicrographs of mice kidney stained with hematoxylin and eosin. Sham (a), sham + simvastatin (b), CLP (c), and CLP + simvastatin (d). Tubular vacuolization (arrow), glomerular cell proliferation (asterisk), increased peri-capsular stroma (star). Bar 50 *μ*m.

**Figure 3:**
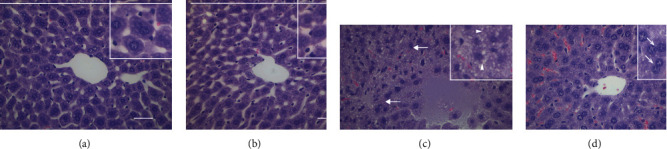
Representative histology photomicrographs of mice liver stained with hematoxylin and eosin. Sham (a), sham + simvastatin (b), CLP (c), and CLP + simvastatin (d). Intense vacuolization of the centrilobular region (thick arrow), hepatocytes vacuolization (arrowhead, insert), and hepatocyte showing binucleation (thin arrow, insert). Bar 100 *μ*m.

Sepsis was induced through CLP, and sham-operated animals were used as controls. Initially, we demonstrated that a single dose treatment with simvastatin (2 mg/kg b.w., intravenously) was able to improve renal function (Figure 1(b)) and to reduce hepatic dysfunction (Figure 1(a)). The histology of the kidney and liver of the sham and sham plus simvastatin animals showed preserved morphological structures, within the normal range. The renal evaluation of the CLP group showed tubular vacuolization and increased glomerular cellularity. The CLP plus simvastatin group demonstrated less cellular damage, with a reduction in tubular vacuolization and glomerular cellularity (Figure 2). The hepatic assessment of the CLP group showed a significant increase in hepatocyte vacuolization and centrilobular vein congestion. The CLP plus simvastatin group showed better tissue organization, with a reduction in hepatocyte vacuolization and increase in the number of binucleation (Figure 3). We also provide a table displaying the liver and kidney alterations (Table 1).

**Figure 4:**
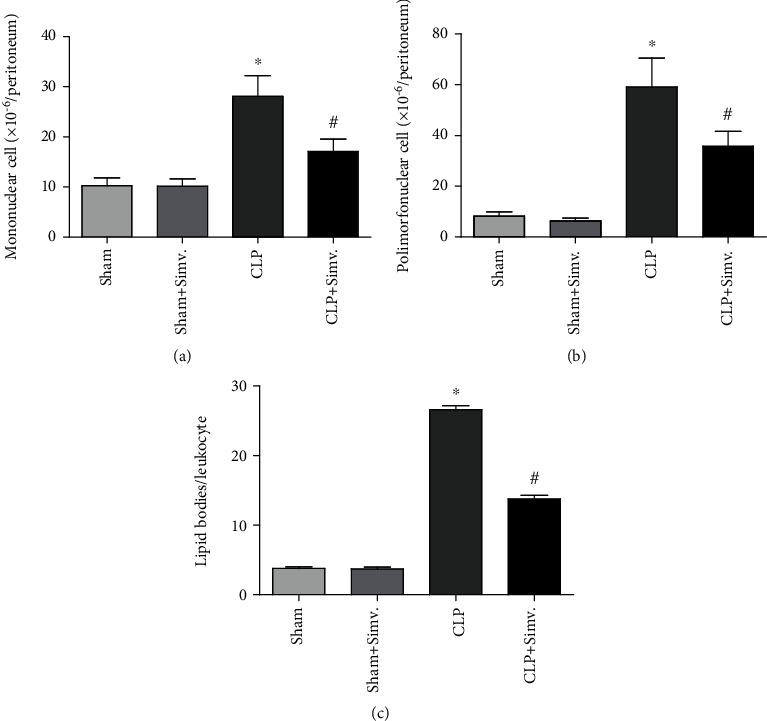
Representative images of lipid bodies staining. The images were captured at 1000x in a light microscope denoting black dots in the cells called lipid bodies.

**Figure 5:**
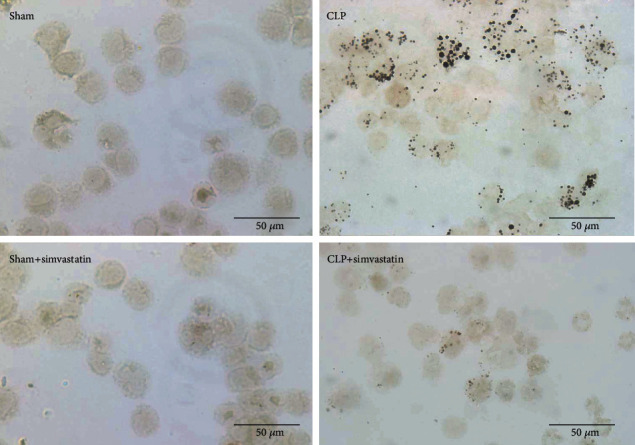
Analysis of cell migration on peritoneal lavage fluid samples of SW submitted to CLP. SW mice submitted to CLP, treated or untreated with simvastatin, had the peritoneum lavage collected 24 hours after sepsis induction for mononuclear (a), neutrophil (b), and lipid body (c) counts. Lipid bodies were enumerated in 50 cells of each animal. Data represented as mean ± SEM of at least 6 animals. ^∗^p < 0.05, control vs. CLP + vehicle; ^#^*p* < 0.05, CLP + vehicle vs. CLP + simvastatin.

Inflammatory parameters were also analyzed in the animals treated with simvastatin. As shown in Figure 4, simvastatin treatment was able to significantly reduce cell migration into the peritoneum. Numbers of both mononuclear cells (Figure 4(a)) and neutrophils (Figure 4(b)) were reduced in the site of inflammation 24 hours after sepsis induction. We also analyzed the effect of simvastatin on cell activation through lipid body quantification. Interestingly, we saw a reduction of lipid body numbers in leukocytes recovered from the peritoneal lavage of simvastatin-treated animals (Figure 4(c)). Representative images of lipid bodies staining are also shown (Figure 5).

**Figure 6:**
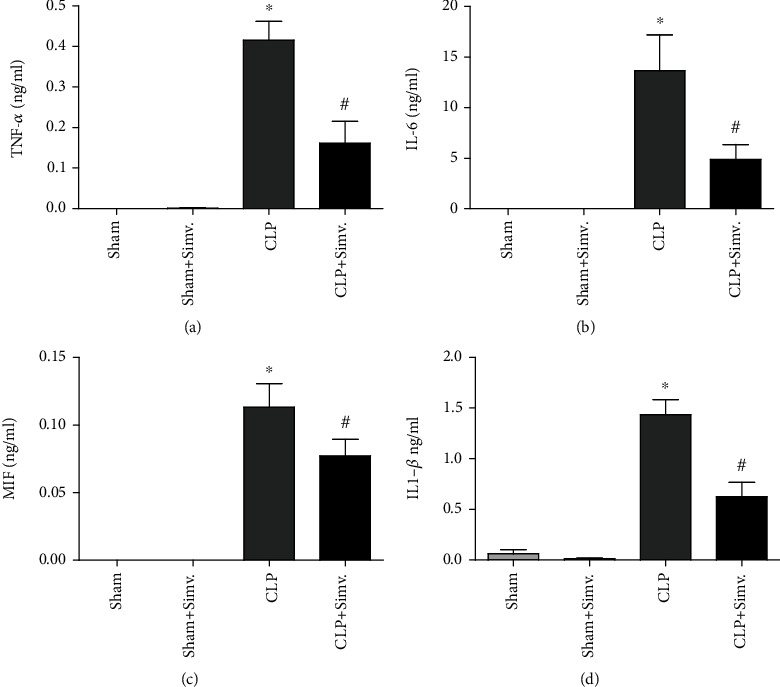
Effect of treatment with simvastatin in the levels of cytokines from peritoneal lavage supernatants of animals submitted to CLP. SW mice submitted to CLP, treated with simvastatin or untreated 6 hours after surgery, had their peritoneal cavity washed 24 hours after sepsis induction. The levels of TNF-*α* (a), IL-6 (b), IL-1*β* (c), and MIF (d) were analyzed. Data represented as mean ± SEM of at least 6 animals. ^∗^*p* < 0.05, control vs. CLP + vehicle; ^#^*p* < 0.05, CLP + vehicle vs. CLP + simvastatin.

CLP induced an increase in the levels of cytokines in peritoneal lavage fluid (Figure 6). As shown in Figure 3, simvastatin treatment was able to reduce the levels of TNF-*α*, IL-6, MIF, and IL-1*β* 24 hours after sepsis induction, indicating an important negative immunomodulary effect of simvastatin in this model.

### 3.2. Simvastatin Decreased Bacterial Load in the Peritoneal Lavage Fluid

**Figure 7:**
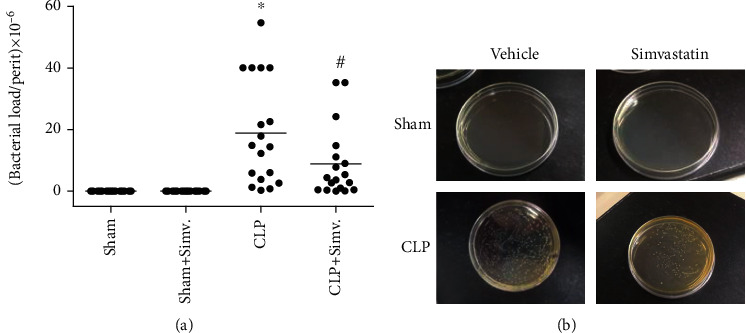
Effect of simvastatin treatment in the bacterial elimination in animals submitted to CLP. SW mice were treated with simvastatin 6 hours after sepsis induction. 24 hours after surgery peritoneal lavage samples were plated for CFU analysis (a). Representative images of CFU Petri dishes per group are shown (b). Data represented as mean ± SEM of at least 6 animals. ^∗^*p* < 0.05, control vs. CLP + vehicle; ^#^*p* < 0.05, CLP + vehicle vs. CLP + simvastatin.

**Figure 8:**
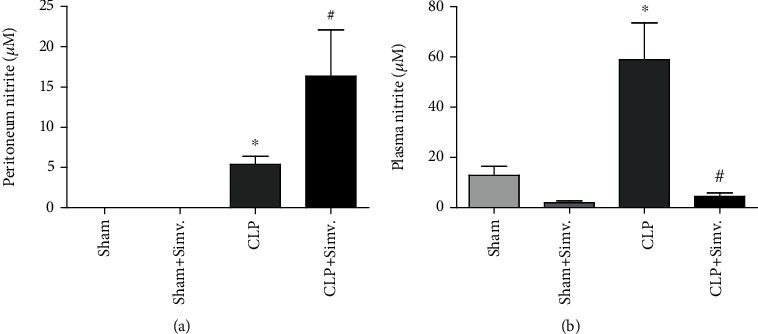
Effect of simvastatin on NO production in animals submitted to CLP. Septic SW mice were treated with simvastatin (2 mg/kg body weight) 6 hours after surgery. 24 hours after sepsis induction, plasma (a) and peritoneal lavage samples (b) were collected for NO quantification. Data represented as mean ± SEM of at least 6 animals. ^∗^*p* < 0.05, control vs. CLP + vehicle, ^#^*p* < 0.05, CLP + vehicle vs. CLP + simvastatin.

To evaluate if simvastatin treatment would affect bacterial clearance, we determined the number of colony forming units in the peritoneal cavity of animals submitted to CLP and treated with simvastatin. As we see in Figure 7, simvastatin treatment was effective in reducing the numbers of colony forming units in the peritoneum of septic animals. As NO is a potent bactericidal agent [23], we investigated the effect of simvastatin on NO production systemically and at the site of infection. As shown in Figure 8(a), simvastatin treatment reduced the levels of NO in the blood while it increased the levels of NO in the peritoneum of animals submitted to CLP (Figure 7(b)).

### 3.3. Simvastatin Decreased Bacterial Load In Vitro

**Figure 9:**
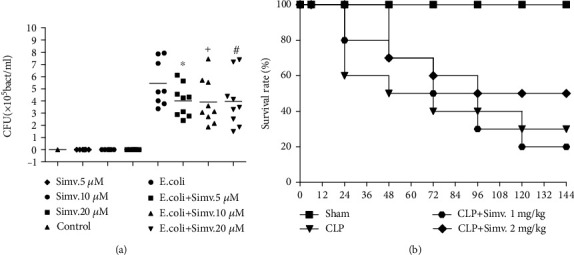
Effect of simvastatin in bacterial elimination by peritoneal macrophages stimulated with *E. coli* and on mortality of animals submitted to CLP. Peritoneal macrophages were pretreated with simvastatin (5 *μ*M, 10 *μ*M, 20 *μ*M), and after 1 hour, they were stimulated with *E. coli* (a). After 30 minutes, the culture supernatants were plated for CFU counting. SW mice were treated with simvastatin (1 mg/kg and 2 mg/kg) 6 hours after surgery. The mortality was observed for 7 days after sepsis induction (b). Data represented as mean ± SEM of at least 6 animals. ^∗^*p* < 0.05, control vs. CLP + vehicle, ^#^*p* < 0.05, CLP + vehicle vs. CLP + simvastatin.

Because of the surprising effect of simvastatin on bacterial load *in vivo*, we evaluated the effect of simvastatin treatment on peritoneal macrophages *in vitro*. Therefore, bacterial colony forming units were counted in the supernatants of a peritoneal macrophage culture incubated with simvastatin and then exposed to *E.coli*.  Figure 9(a) shows that the incubation with simvastatin was able to reduce the CFU numbers in culture supernatant at all concentrations tested.

### 3.4. Effect of Simvastatin on Survival Rate

Once simvastatin successfully improved all inflammatory parameters evaluated in mice submitted to CLP and decreased bacterial load, we investigated its effects on mortality of septic mice. As shown in Figure 9(b), the posttreatment with 2 mg/kg was able to give a 100% of protection in 24 hours after sepsis induction. Although the drug was not able to maintain this protection over 7 days, cognitive test performed with the survivors showed that simvastatin could abrogate memory impairment in septic animals as we have showed previously ([24] and Supplementary Figure 1). The posttreatment with 1 mg/kg gave a lower protection at 24 hours than the 2 mg/kg treatment and had similar survival rate as the CLP + vehicle group. Sham-operated mice had 100% survival rate.

## 4. Discussion

Currently, it is well accepted that sepsis results from an imbalance between proinflammatory reactions, that are responsible for both killing pathogens and tissue damage, and anti-inflammatory reactions, that are responsible for limiting inflammation and increasing vulnerability of the host to infection [25].

Sepsis diagnosis often does not happen in a timely manner, leaving room for the occurrence of dysfunction of multiple organs and system [26]. Therapeutic interventions in the management of sepsis and septic shock represent a clinical challenge, and new approaches and strategies continue to be necessary [27].

Statins, a class of drugs that inhibit HMG-CoA reductase, was introduced during the 1980s in clinical practice, and today is among the most prescribed drugs worldwide. HMG-CoA reductase is an enzyme that participates in the limiting step of cholesterol biosynthesis [28]. Statins have emerged as powerful inhibitors of the inflammatory process, but despite evidence about the potential anti-inflammatory effects of statins, the mechanisms by which they exert these effects are not yet well understood, although the protein-prenylation may have a role on it. Statins have demonstrated promise in the primary and secondary prevention and treatment of patient with sepsis. However, human data remain conflicting; the positive data most frequently come from observational studies, often with inherent healthy user bias [29]. Most of *in vivo* experimental studies have been focused on long- or short-term pretreatment with statins. Thus, in an attempt to better understand the anti-inflammatory actions of statins *in vivo*, we tested posttreatment effect of simvastatin in CLP sepsis [30]. In our study, animals subjected to CLP develop strong inflammatory response, high mortality rate, and cell migration into the site of infection, thus mimicking the profile of septic patients [31]. In order to closely mimic the clinical scenario, we performed CLP in nonisogenic mice and administered the test treatment only after CLP. Alike clinical treatment of sepsis, we did volume replacement and used antibiotics in addition to our tested drug [32].

The liver and kidneys are important organs affected in sepsis, and dysfunction of these organs is associated with high mortality [33, 34]. The animals subjected to CLP showed high levels of oxaloacetic transaminase and creatinine, reflecting liver and kidney dysfunction, respectively. Simvastatin reduced liver damage caused by endotoxemia [35], and we saw the same protection in our model, in addition to a decrease in kidney injury indicated by lower creatinine levels in simvastatin-treated animals. Similar renal protective effect was shown previously by Yasuda et al. [36] [36] and was attributed to a vascular effect of simvastatin.

Statins are capable of reducing the expression of adhesion molecules on monocytes [37] circulating in patients with hypercholesterolemia [38], as well as in endothelial cells [39]. In our study, simvastatin reduced cell accumulation that might reflect a reduced capacity to roll and adhere before transmigration into tissues. Simvastatin effects on leukocyte rolling and adhesion have already been showed, because it reduces leukocyte-endothelial interactions in the cerebral microvasculature of hypertensive rats [40]. Our previous studies showed that statins reverted the decrease in functional capillary density and blocked rolling and adhesion of leukocytes to inflamed endothelium in a model of cerebral malaria [17]. We also revealed that statins diminished microglia activation, lipid peroxidation, and leukocyte-endothelium interactions in the brain vasculature of septic mice [41].

We observed that statins decreased the state of activation of leukocytes recovered in the peritoneal fluid as they had a reduced numbers of lipid bodies in comparison to leukocytes retrieved from septic simvastatin-untreated animals. Lipid bodies are sites of compartmentalization of enzymes forming inflammatory lipid mediators [42] that are increased in sepsis [43]. The decreased numbers of lipid bodies in treated animals might lead to lower production of inflammatory mediators and consequently decreased cell accumulation in the peritoneum helping preventing tissue damage caused by overwhelming inflammation.

Statins improved cardiovascular function and mortality rate in mice submitted to CLP [14, 44], and cerivastatin reduced the release of TNF-*α* and IL-6 in mice after LPS challenge [45]. In sepsis, TNF-*α* is related to organ dysfunction and increased lethality [46, 47], while MIF upregulate TLR-4 and TNF-*α* in macrophages [48, 49]. Also, increased concentrations of MIF were detected in the peritoneal exudate fluid in bacterial peritonitis. Anti-MIF antibody protected mice from lethal peritonitis induced by CLP [50]. IL-6 takes part into acute-phase response [51], and it is an important biomarker of sepsis severity [52]. Our results indicate that simvastatin treatment has an important negative immunomodulatory effect in sepsis since it was able to lower the levels of IL-6, TNF-*α*, IL-1*β*, and MIF in the peritoneal cavity in our model.

NO maintains microcirculation homeostasis by regulating microvascular tone, leukocyte and platelet adhesion, and microvascular permeability [53]. In sepsis, systemic overproduction is harmful causing vasodilation and contributing to haemodynamic instability. However, NO may also act locally as a potent bactericidal agent [23]. Our group has published the enzyme PAF acetylhydrolase (PAF-AH) enhanced bacterial clearance in sepsis model in mice. The levels of NO increased in peritoneal cavity after PAF-AH administration, and PAF-AH treatment did not decrease CFU numbers in inducible nitric oxide synthase- (iNOS-) deficient mice, showing iNOS dependence on more efficient bacterial elimination [54]. We also previously demonstrated the clearance of bacteria involved NO by iNOS production in an ERK-dependent signaling pathway [55]. Simvastatin decreases NO overproduction in a model endotoxin shock in rats [56, 57]. In our work, simvastatin decreased levels of NO in the plasma of animals indicating that the drug may have a beneficial effect in sepsis. Furthermore, simvastatin increased NO production in peritoneum cavity that can contribute to increased bacterial elimination. In fact, simvastatin increased peritoneal bacterial clearance in our model. We believe that the ability of simvastatin to restrain bacterial spreading is due to an increase in local production of NO. According to previous work, statins increase phagocyte extracellular trap formation [58]. However, in our conditions, we were not able to detect enhanced extracellular DNA in peritoneal cavity 24 h after CLP in simvastatin-treated mice (data not shown). Simvastatin was effective in increasing peritoneal macrophage ability to kill bacteria *in vitro*. We suggest simvastatin target peritoneal macrophages increasing their ability to produce NO and to kill bacteria, diminishing bacterial dissemination and further overproduction of inflammatory mediators and endothelial cell activation. The increased peritoneal NO production may be a result of an enhanced local iNOS expression induced by simvastatin treatment. Statins role on the iNOS expression is controversy. Some reports support our view showing statins induce iNOS. Nevertheless, we have to consider there are *in vitro* and *in vivo* reports describing statins inhibit or induce iNOS mRNA and protein expression [59–62]. Therefore, further studies should be made in order to evaluate simvastatin posttreatment cell and tissue specific effect in modulating the balance between eNOS/iNOS activity in sepsis.

It has been shown that pretreatment with statins is capable of increasing survival of animals subjected to CLP and that this increase was accompanied by improvements in cardiovascular functions of these animals [14]; however, statin therapy has no effect on mortality in the overall population of adult septic patients [63]. We have observed a protection at 24 h in simvastatin-treated septic animal, although simvastatin posttreatment has not increased survival rate observed after 7 days. This data resembles the effect on pneumonia where mortality in the simvastatin-receiving cohorts was equivalent to controls [64]. Interestingly, we observed an inhibition of cognitive damage by simvastatin in survivor animals (Supplementary data—figure 1). It is possible that some animals had a hyperinflammatory response, which surpassed the critical threshold leading to death. Nevertheless, if the critical inflammatory threshold was not reached and the animal survived, antimicrobial and anti-inflammatory effects of statins protected the mice from latter consequences such as cognitive damage. Indeed, our group had previously shown that oral treatment with statins reverted neuroinflammation and cognitive decline in a model of intraperitoneal injection of feces [24].

Altogether, our results showed that posttreatment of CLP animals with simvastatin decreased peritoneum cell accumulation and activation, diminished the production of inflammatory mediators, decreased levels of NO in circulation, but increased NO production in the peritoneal cavity reducing bacterial load. MIF secretion seems to have a dynamic kinetics reaching its peak in plasma 8 hours after CLP (Pollak et al.). In addition to that, Calandra et al. demonstrated higher levels of this cytokine in plasma than in peritoneal lavage where it has reached its peak 6 hours after sepsis induction. As seen in our study, MIF levels in peritoneal fluid after sepsis induction are low, probably because we measured it 24 hours after CLP, but still, simvastatin treatments were capable to decrease it. Our data also show that simvastatin is associated with clinical improvement, as the liver and renal functions are improved by simvastatin treatment. Given their pleiotropic effects, statins may represent a useful therapeutic adjunct in the management of sepsis [13]. We suggest that the use of simvastatin as adjunctive therapy for treatment of sepsis should be further investigated.

## Figures and Tables

**Table 1:** 

	Liver		Kidney
	Hepatocyte vacuolization	Hepatocyte binucleation	Tubular vacuolization
Sham	+	+++	+
Sham + simvastatin	+	+++	+
CLP	+++	+	++++
CLP + simvastatin	++	++++	++

## Data Availability

All data used to support the findings of this study are included within the article.

## Supplementary Materials

Supplementary Materials Supplemental Figure 1: simvastatin abrogates memory impairment in septic animals. The animals were submitted to CLP and received simvastatin (2 mg/kg dose) posttreatment. Step-down inhibitory avoidance test was performed to test aversive memory was tested 1.5 (A; short-) and 24 h (B; long term memory) after training by recording the time-to-platform latency (with a cutoff of 180 seconds). Data are expressed as individual values, and horizontal lines represent the mean latency in seconds. ∗*p* < 0.05, *n* = 5 − 17/group

## References

[1] Singer M., Deutschman C. S., Seymour C. W. (2016). The third international consensus definitions for sepsis and septic shock (Sepsis-3). *JAMA*.

[2] Adam N., Kandelman S., Mantz J., Chretien F., Sharshar T. (2014). Sepsis-induced brain dysfunction. *Expert Review of Anti-Infective Therapy*.

[3] Stenkvist B., Bengtsson E., Dahlqvist B., Eriksson O., Jarkrans T., Nordin B. (1982). Cardiac glycosides and breast cancer, revisited. *The New England Journal of Medicine*.

[4] Karasneh R. A., Murray L. J., Cardwell C. R. (2017). Cardiac glycosides and breast cancer risk: a systematic review and meta-analysis of observational studies. *International Journal of Cancer*.

[5] Didkowska J., Wojciechowska U., Manczuk M., Lobaszewski J. (2016). Lung cancer epidemiology: contemporary and future challenges worldwide. *Annals of Translational Medicine*.

[6] Blanco G. (2005). Na,K-ATPase subunit heterogeneity as a mechanism for tissue-specific ion regulation. *Seminars in Nephrology*.

[7] Goldstein J. L., Brown M. S. (1990). Regulation of the mevalonate pathway. *Nature*.

[8] Savas P., Hughes B., Solomon B. (2013). Targeted therapy in lung cancer: IPASS and beyond, keeping abreast of the explosion of targeted therapies for lung cancer. *Journal of Thoracic Disease*.

[9] Biasucci L. M., Biasillo G., Stefanelli A. (2010). Inflammatory markers, cholesterol and statins: pathophysiological role and clinical importance. *Clinical Chemistry and Laboratory Medicine*.

[10] Durant R., Klouche K., Delbosc S. (2004). Superoxide anion overproduction in sepsis: effects of vitamin e and simvastatin. *Shock*.

[11] Hennessy E., Adams C., Reen F. J., O'Gara F. (2016). Is there potential for repurposing statins as novel antimicrobials?. *Antimicrobial Agents and Chemotherapy*.

[12] Jerwood S., Cohen J. (2008). Unexpected antimicrobial effect of statins. *The Journal of Antimicrobial Chemotherapy*.

[13] Kouroumichakis I., Papanas N., Proikaki S., Zarogoulidis P., Maltezos E. (2011). Statins in prevention and treatment of severe sepsis and septic shock. *European Journal of Internal Medicine*.

[14] Merx M. W., Liehn E. A., Janssens U. (2004). HMG-CoA reductase inhibitor simvastatin profoundly improves survival in a murine model of sepsis. *Circulation*.

[15] Ando H., Takamura T., Ota T., Nagai Y., Kobayashi K. (2000). Cerivastatin improves survival of mice with lipopolysaccharide-induced sepsis. *The Journal of Pharmacology and Experimental Therapeutics*.

[16] Lingrel J. B. (2010). The physiological significance of the cardiotonic steroid/ouabain-binding site of the Na,K-ATPase. *Annual Review of Physiology*.

[17] Reis P. A., Estato V., da Silva T. I. (2012). Statins decrease neuroinflammation and prevent cognitive impairment after cerebral malaria. *PLoS Pathogens*.

[18] Kilkenny C., Browne W. J., Cuthill I. C., Emerson M., Altman D. G. (2010). Improving bioscience research reporting: the ARRIVE guidelines for reporting animal research. *PLoS Biology*.

[19] Goncalves-de-Albuquerque C. F., Medeiros-de-Moraes I. M., Oliveira F. M. (2016). Omega-9 oleic acid induces fatty acid oxidation and decreases organ dysfunction and mortality in experimental sepsis. *PLoS One*.

[20] D'Avila H., Melo R. C., Parreira G. G., Werneck-Barroso E., Castro-Faria-Neto H. C., Bozza P. T. (2006). Mycobacterium bovis bacillus Calmette-Guérin induces TLR2-mediated formation of lipid bodies: intracellular domains for eicosanoid synthesis in vivo. *Journal of Immunology*.

[21] Green L. C., Wagner D. A., Glogowski J., Skipper P. L., Wishnok J. S., Tannenbaum S. R. (1982). Analysis of nitrate, nitrite, and [15N]nitrate in biological fluids. *Analytical Biochemistry*.

[22] Carter J. A., Neville B. G., Newton C. R. (2003). Neuro-cognitive impairment following acquired central nervous system infections in childhood: a systematic review. *Brain Research. Brain Research Reviews*.

[23] Wink D. A., Hines H. B., Cheng R. Y. (2011). Nitric oxide and redox mechanisms in the immune response. *Journal of Leukocyte Biology*.

[24] Reis P. A., Alexandre P. C., D'Avila J. C. (2017). Statins prevent cognitive impairment after sepsis by reverting neuroinflammation, and microcirculatory/endothelial dysfunction. *Brain, Behavior, and Immunity*.

[25] Rittirsch D., Flierl M. A., Ward P. A. (2008). Harmful molecular mechanisms in sepsis. *Nature Reviews. Immunology*.

[26] O'Brien J. M., Ali N. A., Aberegg S. K., Abraham E. (2007). Sepsis. *The American Journal of Medicine*.

[27] Iwashyna T. J., Ely E. W., Smith D. M., Langa K. M. (2010). Long-term cognitive impairment and functional disability among survivors of severe sepsis. *JAMA*.

[28] Cannon C. P., Braunwald E., McCabe C. H. (2004). Intensive versus moderate lipid lowering with statins after acute coronary syndromes. *The New England Journal of Medicine*.

[29] Mermis J. D., Simpson S. Q. (2012). HMG-CoA reductase inhibitors for prevention and treatment of severe sepsis. *Current Infectious Disease Reports*.

[30] Holly M. K., Dear J. W., Hu X. (2006). Biomarker and drug-target discovery using proteomics in a new rat model of sepsis-induced acute renal failure. *Kidney International*.

[31] Delano M. J., Ward P. A. (2016). Sepsis-induced immune dysfunction: can immune therapies reduce mortality?. *The Journal of Clinical Investigation*.

[32] Bochud P.-Y., Glauser M. P., Calandra T. (2001). Antibiotics in sepsis. *Intensive Care Medicine*.

[33] Klenzak J., Himmelfarb J. (2005). Sepsis and the kidney. *Critical Care Clinics*.

[34] Maynard N. D., Bihari D. J., Dalton R. N., Beale R., Smithies M. N., Mason R. C. (1997). Liver function and splanchnic ischemia in critically III patients. *Chest*.

[35] Slotta J. E., Laschke M. W., Wang Y., Schilling M. K., Menger M. D., Thorlacius H. (2015). Inhibition of 3-hydroxy-3-methyl-glutaryl-coenzyme A reductase reduces leukocyte recruitment and hepatocyte apoptosis in endotoxin-induced liver injury. *Journal of Investigative Medicine*.

[36] Yasuda H., Yuen P. S., Hu X., Zhou H., Star R. A. (2006). Simvastatin improves sepsis-induced mortality and acute kidney injury via renal vascular effects. *Kidney International*.

[37] Terblanche M., Almog Y., Rosenson R. S., Smith T. S., Hackam D. G. (2007). Statins and sepsis: multiple modifications at multiple levels. *The Lancet Infectious Diseases*.

[38] Rezaie-Majd A., Prager G. W., Bucek R. A. (2003). Simvastatin reduces the expression of adhesion molecules in circulating monocytes from hypercholesterolemic patients. *Arteriosclerosis, Thrombosis, and Vascular Biology*.

[39] Prasad R., Giri S., Nath N., Singh I., Singh A. K. (2005). Inhibition of phosphoinositide 3 kinase-Akt (protein kinase B)-nuclear factor-kappa B pathway by lovastatin limits endothelial-monocyte cell interaction. *Journal of Neurochemistry*.

[40] Freitas F., Estato V., Reis P. (2017). Acute simvastatin treatment restores cerebral functional capillary density and attenuates angiotensin II-induced microcirculatory changes in a model of primary hypertension. *Microcirculation*.

[41] Mishra N. K., Peleg Y., Cirri E. (2011). FXYD proteins stabilize Na,K-ATPase: amplification of specific phosphatidylserine-protein interactions. *The Journal of Biological Chemistry*.

[42] Bozza P. T., Magalhaes K. G., Weller P. F. (2009). Leukocyte lipid bodies - biogenesis and functions in inflammation. *Biochimica et Biophysica Acta*.

[43] Bozza P. T., Bandeira-Melo C. (2005). Mechanisms of leukocyte lipid body formation and function in inflammation. *Memórias do Instituto Oswaldo Cruz*.

[44] Merx M. W., Liehn E. A., Graf J. (2005). Statin treatment after onset of sepsis in a murine model improves survival. *Circulation*.

[45] Chaudhry M. Z., Wang J. H., Blankson S., Redmond H. P. (2008). Statin (cerivastatin) protects mice against sepsis-related death via reduced proinflammatory cytokines and enhanced bacterial clearance. *Surgical Infections*.

[46] Gaur U., Aggarwal B. B. (2003). Regulation of proliferation, survival and apoptosis by members of the TNF superfamily. *Biochemical Pharmacology*.

[47] Gordon A. C., Lagan A. L., Aganna E. (2004). TNF and TNFR polymorphisms in severe sepsis and septic shock: a prospective multicentre study. *Genes and Immunity*.

[48] Roger T., David J., Glauser M. P., Calandra T. (2001). MIF regulates innate immune responses through modulation of Toll-like receptor 4. *Nature*.

[49] Roger T., Glauser M. P., Calandra T. (2016). Macrophage migration inhibitory factor (MIF) modulates innate immune responses induced by endotoxin and Gram-negative bacteria. *Journal of Endotoxin Research*.

[50] Calandra T., Echtenacher B., Roy D. L. (2000). Protection from septic shock by neutralization of macrophage migration inhibitory factor. *Nature Medicine*.

[51] Leon L. R., White A. A., Kluger M. J. (1998). Role of IL-6 and TNF in thermoregulation and survival during sepsis in mice. *The American Journal of Physiology*.

[52] Hack C. E., De Groot E. R., Felt-Bersma R. J. (1989). Increased plasma levels of interleukin-6 in sepsis. *Blood*.

[53] Trzeciak S., Cinel I., Dellinger R. P. (2008). Resuscitating the microcirculation in sepsis: the central role of nitric oxide, emerging concepts for novel therapies, and challenges for clinical trials. *Academic Emergency Medicine*.

[54] Teixeira-da-Cunha M. G., Gomes R. N., Roehrs N. (2013). Bacterial clearance is improved in septic mice by platelet-activating factor-acetylhydrolase (PAF-AH) administration. *PLoS One*.

[55] Gomes R. N., Teixeira-Cunha M. G., Figueiredo R. T. (2013). Bacterial clearance in septic mice is modulated by MCP-1/CCL2 and nitric oxide. *Shock*.

[56] Chen C. H., Lee R. P., Wu W. T., Liao K. W., Hsu N., Hsu B. G. (2007). Fluvastatin ameliorates endotoxin induced multiple organ failure in conscious rats. *Resuscitation*.

[57] Greenwood J., Mason J. C. (2007). Statins and the vascular endothelial inflammatory response. *Trends in Immunology*.

[58] Chow O. A., von Kockritz-Blickwede M., Bright A. T. (2010). Statins enhance formation of phagocyte extracellular traps. *Cell Host & Microbe*.

[59] Araki S., Dobashi K., Asayama K., Shirahata A. (2009). Simvastatin enhances induction of inducible nitric oxide synthase in 3T3-L1 adipocytes. *Free Radical Research*.

[60] Habara K., Hamada Y., Yamada M. (2008). Pitavastatin up-regulates the induction of iNOS through enhanced stabilization of its mRNA in pro-inflammatory cytokine-stimulated hepatocytes. *Nitric Oxide*.

[61] Parihar S. P., Guler R., Brombacher F. (2019). Statins: a viable candidate for host-directed therapy against infectious diseases. *Nature Reviews. Immunology*.

[62] Ye Y., Martinez J. D., Perez-Polo R. J., Lin Y., Uretsky B. F., Birnbaum Y. (2008). The role of eNOS, iNOS, and NF-kappaB in upregulation and activation of cyclooxygenase-2 and infarct size reduction by atorvastatin. *American Journal of Physiology. Heart and Circulatory Physiology*.

[63] Pasin L., Landoni G., Castro M. L. (2013). The effect of statins on mortality in septic patients: a meta-analysis of randomized controlled trials. *PLoS One*.

[64] Boyd A. R., Hinojosa C. A., Rodriguez P. J., Orihuela C. J. (2012). Impact of oral simvastatin therapy on acute lung injury in mice during pneumococcal pneumonia. *BMC Microbiology*.

