# Supplementary material for: Simvastatin Posttreatment Controls Inflammation and Improves Bacterial Clearance in Experimental Sepsis
Source: Mediators Inflamm. 2020 Oct 14;2020:1839762. doi: 10.1155/2020/1839762 (PMC7582071; doi:10.1155/2020/1839762)
Supplement: Supplementary Materials — Supplemental Figure 1: simvastatin abrogates memory impairment in septic animals. The animals were submitted to CLP and received simvastatin (2 mg/kg dose) posttreatment. Step-down inhibitory avoidance test was performed to test aversive memory was tested 1.5 (A; short-) and 24 h (B; long term memory) after training by recording the time-to-platform latency (with a cutoff of 180 seconds). Data are expressed as individual values, and horizontal lines represent the mean latency in seconds. ∗p < 0.05, n = 5 − 17/group [file 1839762.f1.pdf]

HMG-CoA reductase inhibitor simvastatin post-treatment improves organ dysfunction, inflammatory profile, and bacterial clearance after the onset of sepsis

Flora Magno de Jesus Oliveira[1#], Cassiano Felippe Gonçalves-de-Albuquerque[1,2,#], Isabel Matos Medeiros de Moraes[1], Patrícia Alves Reis[1], Vinicius Novaes Rocha[3], Patrícia Torres Bozza[1], Adriana Ribeiro Silva[1] & Hugo Caire de Castro Faria Neto[1]*

[#] **The first two authors contributed equally to this article.**

[1]Laboratório de Imunofarmacologia, Instituto Oswaldo Cruz, Fiocruz, Rio de Janeiro, RJ, Brazil.

[2]Laboratório de Imunofarmacologia, Instituto Biomédico, Universidade Federal do Estado do Estado do Rio de Janeiro, Brazil

[3]Laboratório de Patologia e Histologia Veterinária, Departamento de Medicina Veterinária, Universidade Federal de Juiz de Fora, Brazil

***Corresponding authors:** Hugo Caire Castro Faria Neto, Fundação Oswaldo Cruz, Instituto Oswaldo Cruz - Fiocruz, Pavilhão Ozório de Almeida, Laboratório de Imunofarmacologia, Av Brasil 4365, Rio de Janeiro – RJ – Brazil, CEP 21040-900. Tel: 55-21-25621311 fax 55-21-25621410, e-mail: hugocfneto@gmail.com and Cassiano Felippe Gonçalves-de-Albuquerque, Universidade Federal do Estado do Estado do Rio de Janeiro, Laboratório de Imunofarmacologia, Frei Caneca 94, Centro, Rio de Janeiro – RJ – Brazil, CEP 20211-010. Tel: 55-21- 2531-7711, e-mail: cassiano.albuquerque@unirio.br

Supplemental figure 1 legend: Simvastatin abrogates memory impairment in septic animals. The animals were submitted to CLP and received simvastatin (2mg/Kg dose) post treatment. Step-down inhibitory avoidance test was performed to test aversive memory was tested 1.5 (A; short-) and 24 h (B; long-term memory) after training by recording the time-to-platform latency (with a cut-

off of 180 seconds). Data are expressed as individual values, and horizontal lines represent the mean latency in seconds. *p<0.05, n= 5-17/group.

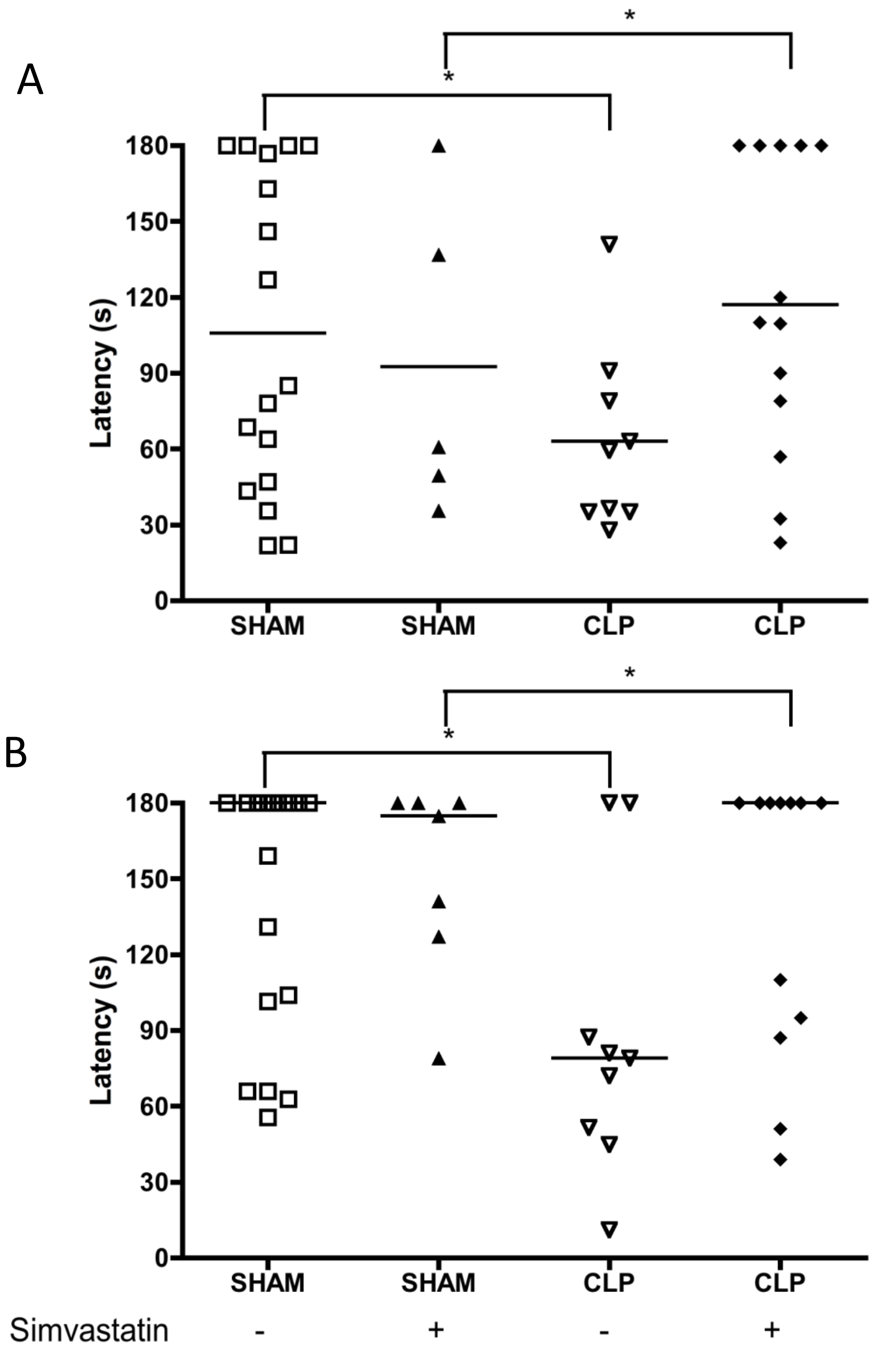

Supplemental Figure 1
